# Clinical Practice: Evidence-Based Recommendations for the Treatment of Cervical Dystonia with Botulinum Toxin

**DOI:** 10.3389/fneur.2017.00035

**Published:** 2017-02-24

**Authors:** Maria Fiorella Contarino, Joost Van Den Dool, Yacov Balash, Kailash Bhatia, Nir Giladi, Johannes H. Koelman, Annemette Lokkegaard, Maria J. Marti, Miranda Postma, Maja Relja, Matej Skorvanek, Johannes D. Speelman, Evelien Zoons, Joaquim J. Ferreira, Marie Vidailhet, Alberto Albanese, Marina A. J. Tijssen

**Affiliations:** ^1^Department of Neurology, Haga Teaching Hospital, The Hague, Netherlands; ^2^Department of Neurology, Leiden University Medical Centre, Leiden, Netherlands; ^3^Department of Neurology AB 51, University Medical Centre Groningen, Groningen, Netherlands; ^4^ACHIEVE Centre of Expertise, Faculty of Health, Amsterdam University of Applied Sciences, Amsterdam, Netherlands; ^5^Movement Disorders Unit of the Department of Neurology, Tel Aviv Sourasky Medical Center, Tel Aviv, Israel; ^6^Sackler Faculty of Medicine, Tel Aviv University, Tel Aviv, Israel; ^7^Sobell Department, Institute of Neurology, National Hospital for Neurology, University College London, London, UK; ^8^Department of Neurology/Clinical Neurophysiology, Academic Medical Center, Amsterdam, Netherlands; ^9^Department of Neurology, Copenhagen University Hospital Bispebjerg, Copenhagen, Denmark; ^10^Department of Neurology, Hospital Clinic i Universitari, Institut D’Investigacio Biomedica August Pi i Sunyer (IDIBAPS), CIBERNED, Barcelona, Spain; ^11^Movement Disorders Center, Department of Neurology, Clinical Medical Center School of Medicine, Zagreb University, Zagreb, Croatia; ^12^Department of Neurology, P. J. Safarik University, Kosice, Slovakia; ^13^Department of Neurology, University Hospital of L. Pasteur, Kosice, Slovakia; ^14^Clinical Pharmacology Unit, Faculty of Medicine, Instituto de Medicina Molecular, University of Lisbon, Lisbon, Portugal; ^15^Sorbonne University, UPMC Paris-6, Paris, France; ^16^Brain and Spine Institute – ICM, Centre for Neuroimaging Research – CENIR, UPMC UMR 1127, Paris, France; ^17^INSERM U 1127, Paris, France; ^18^CNRS UMR 7225, Team Control of Normal and Abnormal Movement, Paris, France; ^19^Department of Neurology, Salpêtriere Hospital, AP-HP, Paris, France; ^20^Department of Neurology, Humanitas Research Hospital, Milano, Italy; ^21^Department of Neurology, Università Cattolica del Sacro Cuore, Milano, Italy

**Keywords:** botulinum toxin, cervical dystonia, recommendations, efficacy, side effects, treatment strategy

## Abstract

Cervical dystonia (CD) is the most frequent form of focal dystonia. Symptoms often result in pain and functional disability. Local injections of botulinum neurotoxin are currently the treatment of choice for CD. Although this treatment has proven effective and is widely applied worldwide, many issues still remain open in the clinical practice. We performed a systematic review of the literature on botulinum toxin treatment for CD based on a question-oriented approach, with the aim to provide practical recommendations for the treating clinicians. Key questions from the clinical practice were explored. Results suggest that while the beneficial effect of botulinum toxin treatment on different aspects of CD is well established, robust evidence is still missing concerning some practical aspects, such as dose equivalence between different formulations, optimal treatment intervals, treatment approaches, and the use of supportive techniques including electromyography or ultrasounds. Established strategies to prevent or manage common side effects (including excessive muscle weakness, pain at injection site, dysphagia) and potential contraindications to this treatment (pregnancy and lactation, use of anticoagulants, neurological comorbidities) should also be further explored.

## Introduction

Cervical dystonia (CD) is the most frequent form of focal dystonia, with an overall prevalence of 4.98/100,000 in Europe (1). CD is characterized by abnormal postures of head and neck that can considerably impair activities of daily living (ADL), with pain occurring in 43.1% of patients (2). Mood disorders, including anxiety and depression, are frequently present (3, 4).

Oral medication has a limited role. Trihexyphenidyl is classically proposed, but the tolerance profile is low (5). Benzodiazepines, especially diazepam and clonazepam, mainly reduce dystonia-related pain, anxiety, and possibly dystonic tremor (6). Tetrabenazine, although possibly effective (7), is limited by the frequent side effect of depression and parkinsonism. Evidence on the effectiveness of allied care treatments, including physiotherapy and cognitive behavioral therapy, is scanty (8). In those with unsatisfactory botulinum neurotoxin (BoNT) effect, surgery may be considered. Peripheral surgery, such as selective peripheral denervation, can provide improvement in about two-thirds of cases, with frequent relapses and is now rarely performed (9, 10). Deep brain stimulation (DBS) of the globus pallidus pars interna appears to be a better choice, despite potential severe complications (11). Alternative DBS targets, such as the subthalamic nucleus, need further investigation (12).

Local injections of BoNT are currently the treatment of choice for CD. By binding to peripheral cholinergic nerve endings in the neuromuscular junction, BoNT decreases the release of acetylcholine at the motor neuron in the synaptic cleft, thus blocking neuromuscular transmission and provoking muscle weakness (13).

Botulinum neurotoxin type A is the most frequently used; type B is only proposed in selected cases.

Although BoNT treatment is widely applied worldwide, many questions remain open in clinical practice.

Some aspects of this treatment have been largely explored in the literature, and robust evidence is available. Other aspects still deserve attention and univocal answers and directives are lacking.

In this paper, literature on BoNT treatment for CD was systematically reviewed based on a question-oriented practical approach. The aim was to provide practical recommendations on common issues in clinical practice.

To this end, we reviewed the evidence concerning the comparison of different formulations of BoNT in improving motor symptoms, pain, and quality of life (QoL), also in relation to the dosage conversion ratio, which is a long debated topic.

Another common issue in the daily practice, which demands stronger evidence is how to prevent and manage side effects and complications, including the formation of neutralizing antibodies (NAB) and treatment side effects such as dysphagia, neck muscle paresis, or pain at injection site.

Due to the nature of the treatment itself, which involves intramuscular injections and a neurochemical denervation, questions may arise concerning potential contraindications such as the use of anticoagulants or the presence of concomitant neuromuscular disorders, in addition to pregnancy and lactation.

We finally explored issues related to the optimization of the treatment, including the optimal initial dose of BoNT, and whether injection strategy can be improved by applying multiple injection points instead of single injection points or by using neurophysiological techniques or associated physiotherapy. These topics have been touched upon in some studies, but the use of different methodologies, protocols, and sometimes patients’ populations makes it difficult to directly compare the results.

## Methods

The aim of this manuscript was to provide a literature review focused on some specific question arising from the clinical practice.

A structured literature review was conducted, by using appropriate keywords covering the topic of BoNT treatment for CD. A language restriction to English, French, German, and Dutch was applied. All kind of studies were reviewed and studies carried out before 1980 were excluded.

### Information Sources

Three databases were searched: Medline and Embase using the Ovid interface, and the Cochrane library.

### Selection of Papers

In all three databases, we identified systematic reviews, randomized controlled trials (RCTs), health economic evaluation studies, and, in both Medline and Embase, also observational studies. The complete search strategy is reported in File S1 in Supplementary Material.

### Review Method

All the papers were screened for topic appropriateness on abstract basis by two independent reviewers with successive agreement on discrepancies. Papers were then assigned to different coauthors according to predefined key clinical questions.

To assess the quality of the published studies, the classification scheme for level of evidence and the level of recommendation of the American Academy of Neurology was used (14) (File S2 in Supplementary Material). The recommendation level is reported for each statement.

## Results

### Effect of Different BoNT Formulations on CD (Table 1)

**Table 1:** **Effect of different formulations of BoNT on CD**.

Question	Answer	Level of recommendation
Is abobotulinumtoxinA effective in improving CD?	Yes	A

Is incobotulinumtoxinA effective in improving CD?	Yes	B

Is onabotulinumtoxinA effective in improving CD?	Yes	A

Is rimabotulinumtoxinA effective in improving CD?	Yes	A

Does BoNT-A treatment improve quality of life?	Yes	B

Does BoNT-A reduce pain associated with CD?	Yes	A

Do BoNT-A and BoNT-B have a comparable effect and duration of effect on dystonia?	Yes	A

Do BoNT-A and BoNT-B have the same rate of side effects?	No (side effects are more frequent with BoNT-B)	B

What is the conversion ratio of onabotulinumtoxinA to abobotulinumtoxinA?	1 IU to 3 IU 1 IU to 2.5 IU	A B

What is the conversion ratio of onabotulinumtoxinA to incobotulinumtoxinA?	1 IU to 1 IU	B

*BoNT, botulinum neurotoxin; CD, cervical dystonia*.

Three BoNT-A products are commercially available: onabotulinumtoxinA (Botox^®^, Allergan), abobotulinumtoxinA (Dysport^®^, Ipsen), and incobotulinumtoxinA (Xeomin^®^, Merz). These products differ concerning the added preservatives, the toxin solubility, and the relative potencies. Only one type of BoNT-B is available: rimabotulinumtoxinB (Neurobloc^®^/Myobloc^®^, Elan Pharma).

#### Are the Different Formulations of BoNT-A and BoNT-B Effective in Improving CD?

Several RCTs showed that onabotulinumtoxinA, abobotulinumtoxinA, and rimabotulinumtoxinB are effective in reducing dystonia when compared to placebo (15–25).

One RCT showed that incobotulinumtoxinA (at both doses of 120 IU and 240 IU) significantly improved Toronto Western Spasmodic Torticollis Rating Scale (TWSTRS)-total scores compared to placebo in 233 CD patients (26) and that improvement of mean TWSTRS-total (*p* < 0.001) and severity score (*p* < 0.016) persisted after repeated injection (up to 5) (27).

##### Conclusion and Recommendations

There is class I evidence that the three BoNT-A and the BoNT-B formulations significantly improve dystonia in CD. The recommendation level is A for abobotulinumtoxinA, onabotulinumtoxinA, and rimabotulinumtoxinB, and level B for incobotulinumtoxinA (26).

#### Does BoNT-A Treatment Improve QoL?

In a double-blind RCT, treatment with 500 IU of abobotulinumtoxinA produced significantly greater improvements than placebo in Physical Functioning, Role Physical, Bodily Pain, General Health, and Role Emotional domains of the SF-36 (*p* ≤ 0.03) (28).

##### Conclusion and Recommendations

There is class I evidence that BoNT-A improves QoL in CD (level B).

#### Does BoNT-A Reduce Pain Associated with CD?

In five RCTs with a total of 162 CD patients, 71% of the patients treated with onabotulinumtoxinA and abobotulinumtoxinA reported pain reduction compared with 12% of the patients in the placebo group (*p* < 0.00001) (29). Pain was also improved with incobotulinumtoxinA in both single-set injections and long-term treatment (26, 27).

##### Conclusion and Recommendations

There is class I evidence that BoNT-A reduces pain symptoms in CD (level A).

#### Do BoNT-A and BoNT-B Have Comparable Effects?

In two RCTs (30, 31), no difference was found in the size and duration of effect on the total TWSTRS score and sub-scores (dystonia severity, limitations, and pain score) between BoNT-A and BoNT-B. Dry mouth and swallowing difficulties were more common with BoNT-B (30, 32).

##### Conclusion and Recommendations

Botulinum neurotoxin-A and BoNT-B have a comparable effect and duration of effect (level A).

Side effects are more frequent with BoNT-B (class I evidence, level B).

#### What Is the Conversion Ratio of Different Formulations of BoNT-A?

The conversion factor between the different formulations is still a matter of discussion.

##### OnabotulinumtoxinA vs. AbobotulinumtoxinA

LD50 tests have shown 1:1 potency ratio of incobotulinumtoxinA vs. onabotulinumtoxinA (33), and 2.3:1 of abobotulinumtoxinA vs. onabotulinumtoxinA. These data however cannot be directly translated into the clinical practice (34).

In a retrospective study, changing from onabotulinumtoxinA to abobotulinumtoxinA with a conversion rate of 1:2 resulted in a tendency toward higher efficacy but more adverse events (35). At 6.5 years follow-up, the doses had been reduced, and the median dose conversion ratio had decreased to 1:1.7.

In a double-blind study, 79 healthy controls were randomized into 18 groups, receiving different doses and concentrations of onabotulinumtoxinA or abobotulinumtoxinA (36). Both toxins caused a comparable, significant decline in the compound muscle action potential (CMAP). A statistical model with CMAP data indicated a bioequivalence of 1 IU onabotulinumtoxinA:1.57 IU abobotulinumtoxinA and a maximum dose-equivalence ratio of 1:3.

In a comparative clinical study, 73 CD patients were randomized for onabotulinumtoxinA or abobotulinumtoxinA with a dose ratio of 1:3 IU (37). The improvement of Tsui score, the duration of effect, and the rate of side effects were comparable.

Two different conversion factors (1:3 and 1:4) between onabotulinumtoxinA and abobotulinumtoxinA were tested in a double-blind randomized three-period crossover study in 54 CD patients (38). AbobotulinumtoxinA was significantly more effective than onabotulinumtoxinA in reducing Tsui score, with no significant difference between the two conversion ratios. The adverse events were more frequent in the abobotulinumtoxinA group, but only significantly for the 1:4 conversion.

A recent double-blind, randomized, crossover study using a conversion ratio of 1:2.5 IU showed comparable efficacy and adverse effects (39).

##### Conclusion and Recommendations

It is recommended to use a conversion of 1 IU onabotulinumtoxinA to 3 IU abobotulinumtoxinA (level A) (37, 38), although conversion ratios of 1:2.5 might be equally safe and effective (class I, level B) (39).

##### OnabotulinumtoxinA vs. IncobotulinumtoxinA

In an open label prospective crossover study, 40 patients initially treated with onabotulinumtoxinA were randomly assigned to treatment switch to incobotulinumtoxinA with a 1:1 ratio (33). Inter-injection intervals and treatment duration showed comparable efficacy for at least four injection cycles. Comparable efficacy on TWSTRS and adverse-event profiles for up to 16 weeks were also reported in a randomized, double-blind, parallel-group, non-inferiority trial, with CD patients randomized to incobotulinumtoxinA or onabotulinumtoxinA with the same conversion factor of 1:1 (40).

##### Conclusion and Recommendations

It is recommended to use a conversion of 1:1 IU onabotulinumtoxinA to incobotulinumtoxinA (class I, level B).

### Optimization of BoNT Treatment for CD (Table 2)

**Table 2:** **Optimization of BoNT treatment for CD**.

What is the recommended initial dose for treatment of CD with abobotulinumtoxinA?	500 IU (although other dosages might be used)	A

What is the recommended initial dose for treatment of CD with incobotulinumtoxinA?	120 IU	B

What is the recommended initial dose for treatment of CD with onabotulinumtoxinA?	No recommendation	U

What is the recommended initial dose for treatment of CD with rimabotulinumtoxinB?	2,500 or 5,000 IU 10,000 IU	B A

Can prior polymyographic EMG (pEMG) and EMG guidance improve the treatment outcome in treatment-naïve patients?	Yes	A

Can prior pEMG and EMG guidance improve the treatment outcome in patients with deterioration of treatment effect?	Yes	C

Are multiple-points injections per muscle more effective than single-point injections?	Yes	U

Can additional physiotherapy improve the effect of BoNT treatment?	No (motor improvement as measured by TWSTRS or Tsui score)	C
	Yes (disability and pain and prolongs the effect of BoNT)	U

*BoNT, botulinum neurotoxin; CD, cervical dystonia; EMG, electromyography; TWSTRS, Toronto Western Spasmodic Torticollis Rating Scale*.

#### What Is the Recommended Initial BoNT Dose for Treatment of CD?

According to the respective summary of product characteristics (SPC—last accessed 08/04/2015), the suggested starting total dose is 500 IU in two-three muscles, for abobotulinumtoxinA (SPC last text revision 11/12/2013), and <200 IU (50 IU/injection and maximum 100 IU to the sternocleidomastoid) for onabotulinumtoxinA (SPC, 19/03/2015). For incobotulinumtoxinA, a total dose of 200 IU is mentioned, with doses up to 300 IU allowed (50 IU/injection—SPC, 16/11/2012). For rimabotulinumtoxinB, an initial dose of 5,000 IU may be considered, but a dose of 10,000 IU divided between two and four muscles may be more effective (SPC, 26/02/2014).

In an RCT, 73 patients were randomized into four groups treated with placebo, abobotulinumtoxinA 250, 500, or 1,000 IU, divided between one splenius capitis and the contralateral sternocleidomastoid muscle (21). The greatest improvement was found in the group treated with 1,000 IU, although significantly more side effects were reported. An initial dose of 500 IU abobotulinumtoxinA (divided into 100–200 IU in the sternocleidomastoid muscle, 250–350 IU in the splenius, 100–200 IU in the trapezius, and 100–200 IU in the levator scapulae) significantly improved CD with respect to placebo in another RCT on 68 patients (41). Based on these results, an initial dose of 500 IU abobotulinumtoxinA is suggested. It is worth mentioning, however, that CD could be successfully treated using an average total dose of 200–400 IU abobotulinumtoxinA under electromyography (EMG) guidance, resulting also in fewer side effects (42).

A starting dose of 50–100 IU of onabotulinumtoxinA per muscle, with a maximum dose per session of 280 IU, was used in a study on 32 patients. A documented improvement in both subjective and objective parameters was observed in 75% of patients (43). The mean total doses of original onabotulinumtoxinA injections, reported in 30 studies, as assessed by a systematic review, ranged from 60 to 374 IU in total (44).

In an RCT, both doses of 120 IU and 240 IU incobotulinumtoxinA significantly improved the TWSTRS-total scores compared to placebo in previously treated and treatment-naive subjects, with mild side effects. Initial dose 120 IU of incobotulinumtoxinA has been suggested based on these results (26).

Three double-blind, randomized, placebo-controlled studies (20, 22, 23) have shown that the effect of botulinum toxin B injections in doses of 2,500, 5,000, and 10,000 IU was significantly higher compared to placebo, with the highest clinical effect seen with dose of 10,000 IU as measured by the TWSTRS-total score. The incidence of mild dysphagia was higher in the 10,000 IU group (16, 10, and 27%, respectively, as compared to no patient who received placebo) (20).

##### Conclusion and Recommendations

An initial total dose of 500 IU abobotulinumtoxinA is effective (level A), although other dosages might be used (41, 45).

An initial total dose of 120 IU incobotulinumtoxinA is probably effective (evidence class I, level B) (26).

No clear recommendations can be given on the optimal starting does of onabotulinumtoxinA (level U).

An initial total dose of 2,500 or 5,000 IU rimabotulinumtoxinB (evidence class I, level B) or 10,000 IU (level A) is probably effective.

#### Can Prior Polymyographic EMG (pEMG) and Simultaneous EMG Improve the Treatment Outcome?

In one RCT, 52 CD patients were randomized into a pEMG-group (treated muscles selected based on clinical evaluation and pEMG, and BoNT injected using simultaneous EMG) or control group (muscles selected based solely on clinical examination and injected without EMG) (46). Improvement on the TWSTRS was higher in the pEMG with EMG assistance group (14 vs. 5%).

In a randomized prospective, blinded study on 26 treatment-naive patients, the objectively measured clinical outcome was significantly better when the muscle selection was based on quantitative EMG and treatment was performed with simultaneous EMG, than when treatment was based on clinical judgment alone (47).

Other studies showed that without pEMG, 24–41% of the dystonic muscles were missed, and 25–35% of the injected muscles were misjudged as dystonic (47–49).

A retrospective study explored results of treatment with pEMG in 40 patients with previously unsatisfactory treatment response (50). After 1 year, a significant improvement in both Tsui scores and subjective evaluation was observed. pEMG led to change in injection pattern in 96% of the patients.

In another study, 8/10 CD patients with deterioration of treatment effect, achieved marked improvement (64% on TWSTRS) after pEMG guided injections (51).

The identification of motor endplate zones with high-density surface EMG may help decreasing the BoNT dose by keeping the effect unaltered (52).

##### Conclusion and Recommendations

There is class I evidence that, in treatment-naïve patients, improvements in dystonia and pain are greater if muscles are selected based on a combination of clinical examination and pEMG and injections are performed with EMG guidance (level A) (46, 47).

In patients with deterioration of treatment effect, the use of pEMG and EMG guidance can improve the results (class III, level C) (50, 51).

#### Are Multiple-Points Injections per Muscle More Effective than Single-Point Injections?

No RCTs on this topic were found. A comparative study in 49 patients showed that multiple injections are more effective than a single injection in improving dystonia, pain, posture deformity, range of motion, and activity endurance (53). Experts recommend the administration of one to four injections per muscle, depending on the volume of the muscle (4, 54).

##### Conclusion and Recommendations

There are indications (class III) that multi-point BoNT injections are more effective than single-point BoNT injections (level U).

#### Can Physiotherapy Improve the Effect of BoNT Treatment?

In one single-blind RCT, no significant difference was found between patients randomized to BoNT treatment combined with relaxation therapy alone or with a 12-week physiotherapy program and relaxation therapy (55).

In one crossover RCT on 40 patients, significantly greater reductions in disability in ADL and subjective pain were observed after a 6-week additional physiotherapy, with respect to BoNT treatment alone. In addition, clinical benefit lasted longer and a lower BoNT dose was needed at reinjection. No significant differences were observed on the Tsui scale and TWSTRS (56).

In a case–control open study, 40 patients followed a 4-week physiotherapy program combined with BoNT treatment or BoNT treatment alone. The physiotherapy group showed significantly more improvement on the pain subscale of the TWSTRS, and on some subscales of the SF-36 (57).

##### Conclusion and Recommendation

Adding physiotherapy in combination with BoNT treatment does not produce a greater motor improvement as measured by TWSTRS or Tsui (class II, level C) (55).

Adding physiotherapy to BoNT treatment may improve disability, pain, and prolong the effect of BoNT [class III (4) and IV (57), level U].

### Primary and Secondary Non-Responsiveness (SNR) (Table 3)

**Table 3:** **Primary and SNR**.

Are treatment intervals <12 weeks safe?	Yes (incobotulinumtoxinA) No recommendation (rimabotulinumtoxinB, onabotulinumtoxinA, and abobotulinumtoxinA)	U U

Which treatment strategies are useful in case of non-response to BoNT-A treatment?	Keeping the treatment intervals constant (early detection of SNR)	U
	Repeated plasma exchange (contrasting NAB-induced SNR)	U
	Switching to BoNT-B produces only temporary benefit	U

*BoNT, botulinum neurotoxin; NAB, neutralizing antibodies; SNR, secondary non-responsiveness*.

Primary non-responsiveness to BoNT, defined as lack of treatment effect from the first application, and due to genetically induced resistance (58) or a prior (unnoticed) botulism (59), is exceptional. Technical aspects such as insufficient dosing, errors during drug storage and reconstitution, or improper injection sites could also lead to an initial lack of response, usually amended in successive treatments.

Secondary non-responsiveness is defined as “insufficiently improved posture after three or more unsuccessful injection cycles in CD patient’s previously achieving satisfactory results” (60). SNR concerns around 3–5% of the patients (61).

The formation of NAB, with estimated frequency in CD patients varying from 1.2% (62) to 40% (63), is one of the causes of SNR. NAB have been found in patients treated with onabotulinumtoxinA, abobotulinumtoxinA, and rimabotulinumtoxinB (64). RimabotulinumtoxinB seems more likely to elicit SNR than BoNT-A: antibody-induced therapy failure was shown in 44% of CD patients treated with BoNT-B during a short period (65). The development and titer of NAB does not correlate with the entity of SNR, and there is evidence that the mere detection of NAB does not necessarily indicate the presence of SNR (66, 67). No antibodies are described after treatment with incobotulinumtoxinA in naive CD patients (68, 69), while this has been reported in one patient previously treated with another BoNT (33).

Factors significantly associated with SNR include previous recourse to other therapies such as surgical interventions, physical therapy and neuroleptic use, a higher number of serious adverse events, more frequent treatment interruptions, and higher average BoNT-A doses during the last three injection cycles (67).

#### Are Treatment Intervals <12 Weeks Safe?

No controlled studies have compared the long-term immunogenicity of different BoNT-A.

In a consensus statement, experts recommend that reinjection is left as long as clinically possible, to minimize the chance of antibody responses (4).

The current manufacturer information suggest that the minimal interval between injections should be 10 (SPC onabotulinumtoxinA and incobotulinumtoxinA) to 12 weeks (SPC abobotulinumtoxinA). This information, however, was based on data obtained with the original formulation of onabotulinumtoxinA, which contained a higher protein load (70, 71).

Fixed 3-month intervals may result in a decrease in treatment satisfaction toward the end of the period. Indeed up to 45% of patients indicated a preference for treatment intervals ≤10 weeks (72).

In a trial with incobotulinumtoxinA, where injection sessions were administered at intervals of 6–20 weeks, there were no differences in the tolerability profile in the group of patients injected at 6–14 weeks with respect to the other groups (27).

##### Conclusion and Recommendations

There is only one class I study showing that, with incobotulinumtoxinA, treatment intervals <12 weeks do not increase the risk of developing antibodies. There is insufficient data to recommend or discourage the use of an interval <12 weeks for treatment with rimabotulinumtoxinB, onabotulinumtoxinA, and abobotulinumtoxinA (level U).

#### Which Treatment Strategies Are Useful in Case of SNR to BoNT-A Treatment?

Secondary non-responsiveness develops gradually, starting with a reduced duration of clinical effect and culminating with significant reduction of the maximal effect (73). Therefore, constant treatment intervals and careful scoring of treatment effect may lead to an early detection of SNR (74). However, whether an early detection is useful to prevent the development of SNR and the induction of high titers of NAB is unclear, considering the absence of effective prevention strategies.

Switching from BoNT-A to BoNT-B in patients with SNR due to NAB may initially result in effective treatment; however, most of these patients will eventually develop antibodies to BoNT-B as well (75, 76).

Neutralizing antibodies depletion by repeated plasma exchange in one patient with SNR, allowed recovery of BoNT-A treatment effect (77).

##### Conclusion and Recommendations

It is suggested that keeping the treatment intervals constant may lead to early detection of SNR (level U).

Repeated plasma exchange is possibly effective in contrasting NAB-induced SNR (level U).

Switching to treatment with BoNT-B produces only temporary recovery of effect, often followed by development of antibodies against BoNT-B (level U).

### Management of Side Effects of BoNT Treatment (Table 4)

**Table 4:** **Side effects and contraindications of BoNT treatment for CD**.

What is the most effective to avoid dysphagia?	The additional use of ultrasound may lessen recurrent dysphagia	U

What is the most effective strategy in case of neck muscles paresis?	The use of a soft collar can relieve the symptoms of neck extensor muscles paresis	U

What is the most effective strategy to prevent injection pain?	Skin cooling or local application of anesthetic cream reduce injection pain	U

Is BoNT treatment safe during pregnancy and lactation?	BoNT treatment during pregnancy and lactation is not recommended and should be avoided whenever possible	U

Is BoNT treatment safe for CD patients who use anticoagulants?	The risk of hematoma following BoNT treatment by concomitant use of coumarin derivatives is low	U

Is BoNT treatment safe for CD patients with concomitant neurological comorbidities?	Patients with concomitant impairment of neuromuscular transmission may experience clinical deterioration after BoNT treatment, although in selected cases treatment might be safe and beneficial	U

*BoNT, botulinum neurotoxin; CD, cervical dystonia*.

#### What Is the Most Effective Strategy to Avoid Dysphagia following BoNT Treatment?

Swallowing difficulty is caused by BoNT spreading to the throat muscles. Bilateral sternocleidomastoid injections are more frequently associated with dysphagia (54). Dysphagia is often mild (severe in <5% of the cases), very rarely requires hospitalization or feeding tube, and disappears gradually after 2–3 weeks (54). Dysphagia is relatively common: 7.1% of the patients reported dysphagia after treatment with the original onabotulinumtoxinA, 3.4% with the new generation onabotulinumtoxinA, 19.4% with abobotulinumtoxinA, 12.6% with incobotulinumtoxinA, and 15.6% with rimabotulinumtoxinB (26, 44, 78). Different tendency to spread into surrounding muscles could rely on differences in formulation, size of the protein molecules, or dilution factor, although these results are based on heterogeneous studies in terms of patient selection, dose, and injected muscles (44).

In a study, five CD patients who had reported 34 episodes of dysphagia over 98 EMG-guided injections (34.7%) were treated with additional use of ultrasounds: this resulted in no episodes of dysphagia across 27 injection sessions (79).

##### Conclusion and Recommendations

The additional use of ultrasound may lessen recurrent dysphagia after botulinum treatment (class IV, level U).

#### What Is the Most Effective Strategy in Case of Neck Muscles Paresis following BoNT Treatment?

Weakness of the neck extensors is a common side effect of BoNT treatment in these muscles. The symptoms are usually mild and are generally resolved within a few weeks (54). According to a systematic review, this side effect was reported by 62/339 (18%) of the patients treated with onabotulinumtoxinA or abobotulinumtoxinA, compared with 9/266 (3%) of the patients in the placebo group (80). In an RCT on 233 patients, neck weakness was reported in 6–10% of patients treated with incobotulinumtoxinA 120 or 240 IU, respectively, as compared to 1% of patients treated with placebo (26).

There is no evidence to support the use of a soft collar, although this measure can relieve symptoms of paresis (4).

##### Conclusion and Recommendations

The use of a soft collar can relieve the symptoms of neck extensor muscles paresis (class IV, level U).

#### What Is the Most Effective Strategy to Prevent Pain at the Injection Site?

In RCTs comparing BoNT-A treatment to placebo, injection pain occurs equally in both groups (4). This pain is usually present for only a few days and is rarely a reason to terminate the BoNT-A treatment (54).

Skin cooling (with ethylchloride spray, dry cold, or ice) or local application of anesthetic cream decreases pain associated with limb or facial botulinum injections (81–83). As such, they may be beneficial in patients for the treatment of CD too, although no specific studies were found.

##### Conclusion and Recommendations

Skin cooling or local application of anesthetic cream can reduce injection pain (class IV, level U).

### Contraindications for BoNT Treatment (Table 4)

#### Is BoNT Treatment Safe during Pregnancy and Lactation?

OnabotulinumtoxinA is classified as pregnancy Category C by FDA: “Animal reproduction studies have shown an adverse effect on the fetus and there are no adequate and well-controlled studies in humans, but potential benefits may warrant use of the drug in pregnant women despite potential risks. This drug should be used during pregnancy only if the benefit outweighs the risk to the fetus.” Animal studies have provided no indications of harm during pregnancy with doses of BoNT-A normally used in clinical practice (84).

Results of a survey on 396 doctors showed that a total of 16 pregnant women had been treated with BoNT, primarily in the first trimester. One patient (8.3%) had a miscarriage, while the other patients gave birth to healthy children after full-term pregnancies (85).The overall risk of miscarriage, regardless of the cause, is 15–20% (86). In the literature, up to 25 women are described who have been treated during each stage of pregnancy: two miscarriages were reported in women with previous history of miscarriage; the other cases reported uneventful pregnancy and healthy children (87).

No studies were found on the use of BoNT during lactation. Due to insufficient data, the manufacturers do not recommend using BoNT during lactation, although it seems unlikely that BoNT may enter breast milk (84).

##### Conclusion and Recommendations

Although several cases have been reported of safe use of BoNT during pregnancy, the effect of BoNT on the unborn child has been insufficiently studied in humans; therefore, BoNT treatment during pregnancy is not recommended and should be avoided whenever possible (class IV, level U).

No studies have been conducted on the effect of BoNT on the nursing child; to exclude side effects, BoNT treatment should be avoided during lactation (class IV, level U).

#### Is BoNT Treatment Safe for CD Patients Who Use Anticoagulants?

No reports of complications resulting from the use of coumarin derivatives or non-vitamin K antagonist oral anticoagulants by CD patients treated with BoNT were found. According to the SPC of coumarin derivates, intramuscular injections are discouraged (but not explicitly forbidden) because of the increased risk of hematomas, while no limitation is reported for subcutaneous injections. The incidence of hematoma after BoNT injection was marginally increased in a group of 32 patients treated with phenprocoumon (3%) with respect to 32 control patients (1.8%) (88).

##### Conclusion and Recommendations

The risk of hematoma following BoNT treatment by concomitant use of coumarin derivatives has not been sufficiently studied but seems low (class IV, level U).

#### Is BoNT Treatment Safe for CD Patients with Concomitant Neurological Comorbidities?

Treatment with BoNT may exacerbate symptoms of coexistent neuromuscular diseases (89, 90) or unmask subclinical cases (91, 92). Myasthenia gravis, amyotrophic lateral sclerosis, and Lambert–Eaton diseases are reported as contraindications to BoNTs treatment in the respective SPCs, although cases of safe CD treatment in patients with myasthenia or amyotrophic lateral sclerosis have occasionally been reported (93, 94).

Generalized weakness has been rarely reported after BoNT injections, most frequently in patients treated for spasticity (95, 96).

##### Conclusion and Recommendations

Patients with preexistent impairment of neuromuscular transmission may experience clinical deterioration after BoNT treatment, although in selected cases treatment might be safe and beneficial (class IV, level U).

## General Considerations

Overall, there is a solid bulk of evidence supporting a good beneficial effect of the different formulations of BoNT in the treatment of CD, with a good benefit-to-risk ratio and a sustained effect over time. However, there is still room for strategies to further improve the efficacy and safety of this treatment. Robust evidence is missing concerning some practical aspects, such as treatment approaches, and the use of supportive techniques including EMG or ultrasounds. Existing knowledge often comes from secondary outcome measures in larger studies designed for other research questions. These studies often use variable methods and outcome measures, which makes comparisons difficult. Future studies should focus on these topics, by using standardized approaches and focusing on only one research question.

It has been noticed that the reported results are not always applicable to the daily practice. This may partly be due to the fact that, in the case of BoNT, optimal treatment requires some variability, according to the needs of the patients and to the progression of the symptoms. The design of future studies should also take this aspect into account.

Although the incidence of adverse events related to BoNT injections, including the formation of NAB, is low, there is a need for established strategies to prevent or manage common side effects of this treatment. To this end, multicentre collaborations are warranted in order to be able to collect an informative number of cases.

Some of the main clinical questions, including the dose equivalence between different formulations and the minimum safe treatment intervals, are matter of discussion already for several years. This knowledge gap could only be addressed by research groups willing to engage in well designed and adequately powered clinical studies.

The continuous commitment of clinicians and basic scientist to produce robust evidence concerning these and other open questions arising from the clinical practice, is fundamental to improve QoL of CD patients.

## Author Contributions

All authors made substantial contributions to the conception of the work, drafted sessions of the manuscript or revised it critically for important intellectual content, gave final approval of the version to be published, and agree to be accountable for all aspects of the work.

## Conflict of Interest Statement

JS, YB, MR, JD, and EZ declare no conflict of interest. MC: advisory board: Medtronic and Boston Scientific. Is coinventor on a patent application relevant to deep brain stimulation? Speaking fees: Abbvie, Medtronic, Boston Scientific, and ECMT. KB: receives royalties from publication of Oxford Specialist Handbook of Parkinson’s Disease and Other Movement Disorders (Oxford University Press, 2008) and of Marsden’s Book of Movement Disorders (Oxford University Press, 2012). He receives a stipend as coeditor of Movement disorders Clinical Practice journal. He received honoraria and/or funding for travel to speak at educational meetings/conferences from Teva–Lundbeck, Ipsen, Allergan, and Merz Pharmaceuticals. He has been paid honoraria to be on advisory board for Ipsen and Allergan companies. NG: grants and personal fees from Teva–Lundbeck, IntecPharma, and NeuroDerm; personal fees from Armon Neuromedical Ltd.\Dexel, Monfort, Pharma Two B, UCB, Novartis, Abbvie, Shaier, Genzyme, Dexel, and Sionara; grants, personal fees and other from Lysosomal Therapeutic Inc; outside the submitted work. In addition, NG has a patent concerning parkinsonian monitoring by body fixed sensors of motion and behavior pending. JK: received research and educational grants from Ipsen and Allergan. AL: speaking fee: Ipsen and Nordicinfu Care. Congress participation funded: Abbvie. MM: received speaking fees from Ipsen, Merz, Allergan, and UCB. MP: travel support from Dystonia Foundation. MS: speakers honoraria and compensations for consultations from Abbvie, Actavis, Egis, Krka, Lundbeck, Medtronic, Teva, and UCB. JF: consultancies: GlaxoSmithKline, Novartis, Teva, Lundbeck, Solvay, Abbott, BIAL, Merck-Serono, Merz, Ipsen, and Biogen. Grants: GlaxoSmithKline, Grunenthal, Fundação MSD (Portugal), Teva, MSD, Allergan, Novartis. Other: BIAL, Biogen. MV: advisory board: Merz. AA: speaker’s honoraria from Ipsen, Merz, Medtronic, Boston Scientific, UCB, and Abbvie. MT: received educational grants and national DystonieNet grants from Ipsen, Allergan Pharmaceutics, Merz, Medtronic, and Actelion.

## Abbreviations

ADL, activities of daily living; BoNT, botulinum neurotoxin; CD, cervical dystonia; CMAP, compound muscle action potential; DBS, deep brain stimulation; EMG, electromyography; IU, international units; NAB, neutralizing antibodies; QoL, quality of life; RCTs, randomized controlled trials; SNR, secondary non-responsiveness; SPC, summary of product characteristics; TWSTRS, Toronto Western Spasmodic Torticollis Rating Scale.
