# Supplementary material for: Clinical Practice: Evidence-Based Recommendations for the Treatment of Cervical Dystonia with Botulinum Toxin
Source: Front Neurol. 2017 Feb 24;8:35. doi: 10.3389/fneur.2017.00035 (PMC5323428; doi:10.3389/fneur.2017.00035)
Supplement: Supplementary file 1 [file Table_1.PDF]

Supplementary file 1

Clinical practice: evidence-based recommendations for the treatment of cervical dystonia with botulinum toxin

CONTARINO Maria Fiorella[a,b*], MD, PhD, VAN DEN DOOL Joost[c,d], PT, MSc, BALASH Yacov[e,f], BHATIA Kailash[g], GILADI Nir[e,f], KOELMAN Johannes H.[h], LOKKEGAARD Annemette[i], MARTI Maria J.[j], POSTMA Miranda[h], RELJA Maja[k], SKORVANEK Matej[l,m], SPEELMAN Johannes D.[h], ZOONS Evelien[h], FERREIRA Joaquim J.[n], VIDAILHET Marie[o,p,q], ALBANESE Alberto[r,s], TIJSSEN Marina A.J[c]*.

Search strategy

Date search July 2010, updated in March 2014

MEDLINE

Ovid MEDLINE® In-Process & Other Non-Indexed Citations, Ovid MEDLINE® Daily and Ovid MEDLINE®

1946 to Weekly with Daily & Weekly Update

1	(dystonia/ OR dystonic disorders/ or meige syndrome/ OR Blepharospasm/ OR ((oro?facial or oro?mandibular or facial or hemi?facial or mandibular) adj3 dystoni$).ti,ab. OR ((focal or cervical or cranio?cervical or neck or primary or hand) adj3 dystoni$).ti,ab. OR lepharospasm$.ti,ab. OR (eyelid? adj1 spasm$).ti,ab. OR (writer? adj1 cramp$).ti,ab. OR ((meige? or brueghel?) adj3 syndrome?).ti,ab.)
2	(exp Botulinum Toxins/ OR (botulinum adj3 (toxin? or neurotoxin?)).ti,ab.)
3	(animals.sh not ((humans and animals).sh OR (rat or rats or rattus).ti.))
4	(1 AND 2) NOT 3
5	limit to (yr="1966 -Current" and (dutch or english or french or german))
6	meta analysis.pt. OR (meta-anal$ or metaanal$).af. OR (quantitativ$ adj10 (review$ or overview$)).tw. OR (systematic$ adj10 (review$ or overview$)).tw. OR (methodologic$ adj10 (review$ or overview$)).tw. OR medline.tw. and review.pt. OR (pooled adj3 analy*).tw.
7	randomized-controlled-trial.pt. OR controlled-clinical-trial.pt. OR randomized controlled trial/ OR randomi?ed controlled trial?.tw. OR random-allocation.af. OR double-blind-method.af. OR single-blind-method.af. OR (random adj8 (selection? or sample?)).tw. OR random$.tw.
8	epidemiologic studies/ OR exp case-control studies/ OR exp cohort studies/ OR cross-sectional studies/ OR (case adj3 control).af. OR (cohort adj5 (study or studies or analy$)).af. OR (follow-up adj5 (study or studies)).af. OR (longitudinal or retrospective or prospective or (cross adj5 sectional)).af. OR (observational adj5 (study or studies)).af.

9	exp Health Care Costs/ OR ec.fs. OR (cost$ or risk$).mp. OR estimate$.tw. OR (costs or cost effective or economic).tw. OR Cost-Benefit Analysis/ OR (cost effective$ or sav$).tw. OR (cost effective or cost effectiveness or sensitivity analys$).tw. OR exp "Costs and Cost Analysis"/ OR (cost or costs).tw. OR Cost-Benefit Analysis/ OR (costs or cost effective).tw. OR exp Economics/ OR quality of life/ OR value of life/ OR Quality-adjusted life years/ OR models, economic/ OR markov chains/ OR monte carlo method/ OR decision tree/ OR ec.fs. OR economic$.tw. OR (cost? or costing? or costly or costed).tw. OR (price? or pricing?).tw. OR (pharmacoeconomic? or (pharmaco adj economic?)).tw. OR budget$.tw. OR expenditure$.tw. OR (value adj1 (money or monetary)).tw. OR (fee or fees).tw. OR "quality of life".tw. OR qol$.tw. OR hrqol$.tw. OR "quality adjusted life year$".tw. OR qaly$.tw. OR cba.tw. OR cea.tw. OR cua.tw. OR utilit$.tw. OR markov$.tw. OR monte carlo.tw. OR (decision adj2 (tree$ or analys$ or model$)).tw. OR ((clinical or critical or patient) adj (path? or pathway?)).tw. OR (managed adj2 (care or network?)).tw. OR Economics/ OR "costs and cost analysis"/ OR Cost allocation/ OR Cost-benefit analysis/ OR Cost control/ OR Cost savings/ OR Cost of illness/ OR Cost sharing/ OR "deductibles and coinsurance"/ OR Medical savings accounts/ OR Health care costs/ OR Direct service costs/ OR Drug costs/ OR Employer health costs/ OR Hospital costs/ OR Health expenditures/ OR Capital expenditures/ OR Value of life/ OR exp economics, hospital/ OR exp economics, medical/ OR Economics, nursing/ OR Economics, pharmaceutical/ OR exp "fees and charges"/ OR exp budgets/ OR (low adj cost).mp. OR (high adj cost).mp. OR (health?care adj cost$).mp. OR (fiscal or funding or financial or finance).tw. OR (cost adj estimate$).mp. OR (cost adj variable).mp. OR (unit adj cost$).mp. OR (economic$ or pharmacoeconomic$ or price$ or pricing).tw.
10	5 and 6 (Systematic reviews)
11	5 and 7 (RCT's)
12	5 and 8 (Observational studies)
13	5 and 9 (Health economic studies)

EMBASE

1	exp focal dystonia/ OR ((oro?facial or oro?mandibular or facial or hemi?facial or mandibular) adj3 dystoni$).ti,ab. OR ((focal or cervical or cranio?cervical or neck or primary or hand) adj3 dystoni$).ti,ab. OR blepharospasm$.ti,ab. OR (eyelid? adj1 spasm$).ti,ab. OR (writer? adj1 cramp$).ti,ab. OR ((meige? or brueghel?) adj3 syndrome?).ti,ab.
2	(botulinum toxin/ OR botulinum toxin a/ or botulinum toxin b/ or botulinum toxin e/ or botulinum toxin f/) OR (botulinum adj3 (toxin? or neurotoxin?)).ti,ab.)
3	((animal$ not (human$ and animal$)) or (nonhuman not (human and nonhuman))).sh. OR (rat or rats or rattus).ti.
4	(1 and 2) NOT 3
5	limit 4 to ((dutch or english or french or german) and yr="1980 -Current")
6	meta analysis/ OR "systematic review"/ OR (meta-analy$ or metaanaly$).tw. OR (systematic$ adj4 (review$ or overview$)).tw. OR (quantitativ$ adj5 (review? or overview?)).tw. OR (methodologic adj5 (overview? or review?)).tw. OR (review$ adj3 (database? or medline or embase or cinahl)).tw. OR (pooled adj3 analy$).tw. OR (extensive adj3 review$ adj3 literature).tw. OR ((meta or synthesis or (literature adj8 database?) or extraction).tw. and review.pt.)
7	controlled clinical trial/ or randomized controlled trial/ OR randomization/ OR Major Clinical Study/ OR random$.tw. OR Double Blind Procedure/
8	cohort analysis/ OR longitudinal study/ OR prospective study/ OR retrospective study/ OR comparative study/ OR intermethod comparison/ OR clinical study/ OR case control study/ OR family study/ OR follow up/ OR (cohort adj5 (study or studies or analy*)).tw. OR (case adj5 control).tw. OR (follow-up or followup or follow up).tw. OR (epidemiologic$ adj5 (study or studies)).tw. OR (cross adj5 sectional adj5 (study or studies)).tw. OR (prospect$ adj5 (study or studies)).tw. OR (observational adj5 (study or studies)).tw.
9	exp health economics/ OR exp health care cost/ OR exp quality of life/ OR economic$.tw. OR (cost? or costing? or costly or costed).tw. OR (price? or pricing?).tw. OR (pharmacoeconomic? or (pharmaco adj economic?)).tw. OR budget$.tw. OR expenditure$.tw. OR (value adj1 (money or monetary)).tw. OR (fee or fees).tw. OR "quality of life".tw. OR qol$.tw. OR hrqol$.tw. OR "quality adjusted life year$".tw. OR qaly$.tw. OR cba.tw. OR cea.tw. OR cua.tw. OR utilit$.tw. OR markov$.tw. OR monte carlo.tw. OR (decision adj2 (tree$ or analys$ or model$)).tw. OR ((clinical or critical or patient) adj (path? or pathway?)).tw. OR (managed adj2 (care or network?)).tw. OR Socioeconomics/ OR Cost benefit analysis/ OR Cost effectiveness analysis/ OR Cost of illness/ OR Cost control/ OR Economic aspect/ OR Financial management/ OR Health care cost/ OR Health care financing/ OR Health

	economics/ OR Hospital cost/ OR (fiscal or financial or finance or funding).tw. OR Cost minimization analysis/ OR (cost adj estimate$).mp. OR (cost adj variable$).mp. OR (unit adj cost$).mp. OR exp economic aspect/ OR costs.tw. OR "cost effectiveness analysis"/ OR (randomi?ed or economic).tw. OR (cost effectiveness or cost effective).tw. OR (cost effectiveness or sensitivity analys$).tw. OR "health care cost"/ OR cost.mp. OR costs.tw.
10	5 and 6 (Systematic reviews)
11	5 and 7 (RCTs)
12	5 and 8 (Observational studies)
13	5 and 9 (Health economic studies)

COCHRANE LIBRARY

ID	Search
#1	((orofacial or oro-facial or oromandibular or oro-mandibular or facial or hemifacial or hemi-facial or mandibular) near/3 dystoni*):ti,ab
#2	((focal or cervical or craniocervical or cranio-cervical or neck or primary or hand) near/3 dystoni*):ti,ab
#3	blepharospasm*:ti,ab
#4	(eyelid* near/1 spasm*):ti,ab
#5	(writer* near/1 cramp*):ti,ab
#6	((meige* or brueghel*) near/3 syndrome*):ti,ab
#7	[mh "Dystonic Disorders"]
#8	[mh "Meige Syndrome"]
#9	[mh Blepharospasm]
#10	(#1 or #2 or #3 or #4 or #5 or #6 or #7 or #8 or #9)
#11	(botulinum near/3 (toxin* or neurotoxin*)):ti,ab
#12	[mh ^"Botulinum Toxins"]
#13	#11 or #12
