# Supplementary material for: Clinical Practice: Evidence-Based Recommendations for the Treatment of Cervical Dystonia with Botulinum Toxin
Source: Front Neurol. 2017 Feb 24;8:35. doi: 10.3389/fneur.2017.00035 (PMC5323428; doi:10.3389/fneur.2017.00035)
Supplement: Supplementary file 2 [file Table_2.PDF]

Classification of level of evidence

**Class I.**<br>A randomized, controlled clinical trial of the intervention of interest with masked or objective outcome assessment, in a representative population. Relevant baseline characteristics are presented and substantially equivalent among treatment groups or there is appropriate statistical adjustment for differences.<br>**The following are also required:**<br>**a.** Concealed allocation<br>**b.** Primary outcome(s) clearly defined<br>**c.** Exclusion/inclusion criteria clearly defined<br>**d.** Adequate accounting for dropouts (with at least 80% of enrolled subjects completing the study) and crossovers with numbers sufficiently low to have minimal potential for bias<br>**e.** For noninferiority or equivalence trials claiming to prove efficacy for one or both drugs, the following are also required*<br>**1.** The standard treatment used in the study is substantially similar to that used in previous studies establishing efficacy of the standard treatment (e.g., for a drug, the mode of administration, dose, and dosage adjustments are similar to those previously shown to be effective).<br>**2.** The inclusion and exclusion criteria for patient selection and the outcomes of patients on the standard treatment are substantially equivalent to those of previous studies establishing efficacy of the standard treatment.<br>**3.** The interpretation of the results of the study is based on an observed-cases analysis.
**Class II.**<br>A randomized controlled clinical trial of the intervention of interest in a representative population with masked or objective outcome assessment that lacks one criteria a–e above or a prospective matched cohort study with masked or objective outcome assessment in a representative population that meets b–e above. Relevant baseline characteristics are presented and substantially equivalent among treatment groups or there is appropriate statistical adjustment for differences.
**Class III.** All other controlled trials (including well-defined natural history controls or patients serving as their own controls) in a representative population, where outcome is independently assessed, or independently derived by objective outcome measurement.
**Class IV.** Studies not meeting Class I, II, or III criteria including consensus or expert opinion.

AAN classification of recommendations

(Note that recommendations can be positive or negative)

**A** = **Established** as effective, ineffective, or harmful for the given condition in the specified population. Requires two consistent Class I studies.
B = **Probably** effective, ineffective, or harmful for the given condition in the specified population. Requires one Class I study or two consistent Class II studies.
C = **Possibly** effective, ineffective, or harmful for the given condition in the specified population. Requires one Class II study or two consistent Class III studies.
U = Data is inadequate or conflicting; given current knowledge, **treatment is unproven**. Studies not meeting criteria for Class I through Class III.

Reference:

French, J., and Gronseth, G. (2008). Lost in a jungle of evidence: we need a compass. Neurology 71(20), 1634-1638. doi: 71/20/1634 [pii]; 10.1212/01.wnl.0000336533.19610.1b [doi].
